# Pre-natal and post-natal exposure to respiratory infection and atopic diseases development: a historical cohort study

**DOI:** 10.1186/1465-9921-7-81

**Published:** 2006-05-23

**Authors:** Anne Zutavern, Stephanie von Klot, Ulrike Gehring, Susanne Krauss-Etschmann, Joachim Heinrich

**Affiliations:** 1 GSF- Institut für Epidemiologie, Neuherberg, Germany; 2 Ludwig-Maximilians Universität, Dr. von Haunersches Kinderspital, Munich, Germany; 3 IRAS, Utrecht University, Utrecht, The Netherlands; 4 KKG Pediatric Immune Regulation, GSF- Institut für Epidemiologie, Neuherberg and Ludwig-Maximilians Universität, Dr. von Haunersches Kinderspital, Munich, Germany

## Abstract

**Background:**

According to the hygiene hypothesis, infections in early life protect from allergic diseases. However, in earlier studies surrogate measures of infection rather than clinical infections were associated with decreased frequencies of atopic diseases. Exposure to infection indicating sub-clinical infection rather than clinical infection might protect from atopic diseases.

Objective: to investigate whether exposure to acute respiratory infections within pregnancy and the first year of life is associated with atopic conditions at age 5–14 years and to explore when within pregnancy and the first year of life this exposure is most likely to be protective.

**Methods:**

Historical cohort study: Population level data on acute respiratory infections from the routine reporting system of the former German Democratic Republic were linked with individual data from consecutive surveys on atopic diseases in the same region (n = 4672). Statistical analyses included multivariate logistic regression analysis and polynomial distributed lag models.

**Results:**

High exposure to acute respiratory infection between pregnancy and age one year was associated with overall reduced odds of asthma, eczema, hay fever, atopic sensitization and total IgE. Exposure in the first 9 months of life showed the most pronounced effect. Adjusted odds ratio's for asthma, hay fever, inhalant sensitization and total IgE were statistical significantly reduced up to around half.

**Conclusion:**

Exposure to respiratory infection (most likely indicating sub-clinical infection) within pregnancy and the first year of life may be protective in atopic diseases development. The post-natal period thereby seems to be particularly important.

## Introduction

Environmental factors play an important role in the increasing prevalence of atopic diseases. The observation of an inverse association between family size and hay fever prompted the hygiene hypothesis suggesting that the protective effect of siblings on atopic diseases might be mediated by infections [1]. Many studies confirmed the "sibling effect" [2] and the effect of other indirect measures of infection like day care attendance [3,4]. Furthermore, they pointed to the existence of a highly sensitive phase for protection though infection, which is present in early infancy or even in foetal life [3-8]. Studies measuring the effect of past infection (as against disease) via IgG antibodies observed protective effects of gastro-intestinal viral diseases on atopic outcomes [9,10]. However, studies measuring direct effects of clinical infectious disease on atopic outcomes predominantly found no protective effects [6,9-12].

"Sibling effect", day care attendance and IgG antibodies represent proxy measures for infection overall. However, these measures do not represent clinical infectious disease but additionally indicate sub-clinical infection meaning an immunological reaction of the body without clinical symptoms of disease. We speculated that maybe not clinical infectious disease but infection (including sub-clinical infection) is responsible for the protective effects on atopic conditions. Consequently, a higher exposure to infections in the population (e.g. an epidemic with acute respiratory infections (ARI)) would increase the likelihood of sub-clinical infection (and clinical disease) of the individual and – if acting in a vulnerable period – would be associated with a decreased frequency of atopic conditions in later life.

Thus the objectives of this study were twofold:

- to test whether a high risk of exposure to ARI in pregnancy or early infancy is associated with decreased frequencies of asthma, eczema, hay fever, atopic sensitization and total IgE at age 5–14 years.

- to explore in what period within pregnancy and the first year of life this exposure is most likely to offer protection.

We used routine data on ARI from the routine reporting system of the former German Democratic Republic [13] as a proxy measure for exposure to infections in pregnancy and early infancy and combined them with data from consecutive surveys on atopic diseases.

## Methods

### Study design

This historical cohort study combined exposure information on ARI from a routine reporting surveillance system with subsequent data from 3 cross-sectional studies in the same region. Data on ARI notifications were drawn from monthly surveillance data from the district of Magdeburg [13] located in Saxony-Anhalt within the former German Democratic Republic. Saxony-Anhalt includes the region where the surveys were conducted. In the German Democratic Republic ARI had to be reported to public health authorities. This was not the case in the Federal Republic of Germany. With the German reunification in October 1990 registration of ARI ceased. In 1992–1993, 1995–1996 and 1998–1999 repeated cross-sectional studies on asthma and allergic diseases were conducted in the areas Bitterfeld, Zerbst and Hettstedt in the former East German state of Saxony-Anhalt. All children aged 5–7 (school entrants), 8–10 (third grade) and 11–14 years (sixth grade) in Zerbst and Hettstett and a subset of children of the same age groups in Bitterfeld were invited to participate in interviews, physical examination and blood collection. Children participating in the surveys were born between May 1977 and June 1993. Selection criteria, study population and study design have been described in detail before [14,15]. Response rates ranged between 68.6% and 92.2% depending on survey and study centre [14,15]. Of the 5539 children participating in any survey, 62% children participated in one survey only, 30% in 2 surveys and 7.7% in all three surveys. Children participating in more than one survey were only included once in the current analysis. Informed consent was obtained from the parents of all children. Ethical approval for the study was obtained from the University of Rostock Ethics Committee.

### Questionnaire data

Questionnaires on atopic diseases, child characteristics, lifestyle and environmental factors were filled in by the parents. In children participating in more than one survey age was as age at the latest survey. Asthma, hay fever and eczema were defined as reported lifetime doctor diagnoses of the respective outcomes in any of the questionnaires. High parental education was defined if at least one parent completed high school. Parental allergy was considered positive if at least one parent reported a history of asthma, hay fever or eczema in any survey.

### Atopic sensitization

Total IgE and specific IgE against common inhalant allergens (*dermatophagoides pteronyssinus*, cat, mixed grasses, birch pollen) were determined by standardized methods with CAP-RAST FEARI (Pharmacia Diagnostics, Freiburg, Germany). *Inhalant allergen sensitization *was defined as any measured specific IgE values >= 0.70 kU/l. *Outdoor allergen sensitization *was defined as specific IgE value against mixed grasses or birch pollen >= 0.70 kU/l. *Indoor allergen sensitization *was defined as specific IgE value against cat or house dust mite >= 0.70 kU/l. An arbitrary cut-off of 400 kU/l was chosen to represent elevated *total IgE*. This cut-off level was chosen to identify children at the very high end of total IgE levels while maintaining a large enough group for statistical analysis. Moreover, we chose a full and even number as cut-off point.

### Study population

We restricted the study population to children born before 1990, because there were no reliable data on ARI from October 1990 onwards (due to the reunification of Germany). Therefore only data from children born between May 1977 and December 1989 were included in the analysis (n = 4673).

### Acute respiratory infections (ARI) [13]

The former German Democratic Republic had a state controlled health system with less than 5% private practitioners. Practitioners had to record ARI and other infectious diseases like malaria, toxoplasmosis, pertussis, listeriosis, scabies, mononucleosis, amoeba, typhoid, paratyphoid and tapeworm infections in notification forms that were passed on to public health authorities. Validation studies for the reporting system do not exist. However, due to the state centralised structure of both surveillance system and reporting physicians a high and regionally stable accuracy is likely. No distinction was made between the type of ARI or viral or bacterial origin of ARI. It can be assumed that the majority of infections were viral. For our analysis months within the period from January 1976 to December 1989 with the highest 5% of ARI notifications were considered high exposure months *(high ARI)*. As this cut off was arbitrary we chose a second cut-off of the highest 10% to be able to check for the consistency of the results. For each child it was established in which month of life between the first month of pregnancy and age 1 year it was exposed to high ARI.

### Statistical analysis

For descriptive and logistic regression analyses high ARI was analysed for the following disjoint exposure periods: trimester 1, trimester 2, trimester 3, month of birth, months 0–3, months 3–6, months 6–9 and months 9–12. The periods were defined high ARI if high ARI was present in at least one of the respective months.

Multivariate logistic regression analyses were performed to establish the final models. We aimed to build models with few covariates only. Therefore only factors confounding the association between the exposure variables and the outcome variables were included in the models. Age-group, month of birth, family size, sex, area, parental allergy and parental education were a priori considered as potential confounder variables. Family size was taken as a proxy variable for siblings because direct information about siblings was not available from the questionnaires. Factors were tested individually. Only factors causing a change of more than 10% in the effect (odds ratio) of the main exposure variable were considered to be relevant. The final models included all confounding variables found to be relevant in any of the models. This approach was chosen to minimise the number of variables in the model to make the model more stable.

In the next step, we aimed to examine our data in an explorative way without confinement to the (mostly) three-month periods described above. We used polynomial distributed lags in a multivariate logistic regression model to assess whether it yields similar results as the first approach and can be used to describe the data. We had no hypothesis on the timing of a potential vulnerable period within the pre- and post-natal period. Since an epidemic might have an effect during the months before and after birth, a logistic model was used that included the exposure in all months between 9th months before birth (month of conception) and 12th month after birth. Instead of entering 22 exposure terms into the model we constrained the ß's to follow a polynomial function of degree 6, to control for collinearity and to use less degrees of freedom [16]. This model indicated the nature of the lag structure between epidemics and the occurrence of the outcome. Statistical analysis was performed with STATA 8.0 (Stata Corp., Texas, USA) and SAS version 8 (SAS Institute Inc, Carc, NC).

## Results

### Population characteristics and prevalence of atopic diseases

Data on atopic conditions and IgE results were available for 4672 and 3955 (84.6%) of the 4673 eligible children, respectively. One child was excluded because it had missing data on all atopic conditions measured. Table 1 summarises the characteristics of the population and the prevalence of allergic conditions. The majority of children belonged to the oldest age-group (73.6%) because children that participated in multiple surveys were assigned the age of the last survey. Within the study population asthma had been diagnosed in 2.6% of children, eczema in 11.7% and hay fever in 6.8%. Inhalant sensitization was prevalent in 27.6% and elevated total IgE in 16% of children, respectively.

**Table 1:** Lifetime prevalence of allergic conditions, exposure high ARI and characteristics of the population

	**n/N**	**%**
Asthma	120/4634	2.6
Eczema	547/4668	11.7
Hay fever	316/4668	6.8
Inhalant allergen sensitization ^1 ^*	1030/3737	27.6
Outdoor allergen sensitization ^2 ^*	738/3737	19.8
Indoor allergen sensitization ^3 ^*	631/3915	16.1
Elevated total IgE ^4*^	631/3955	16.0
Exposed to high ARI in period ^5^		
Trimester 1	927/4672	19.8
Trimester 2	989/4672	21.2
Trimester 3	617/4672	13.2
Month of birth	223/4672	4.8
Months 0–3	573/4672	12.3
Months 3–6	653/4672	14.0
Months 6–9	817/4672	17.5
Months 9–12	738/4672	15.8
Area Zerbst	1426/4672	30.5
Bitterfeld	1925/4672	41.2
Hettstett	1321/4672	28.3
Male sex	2464/4672	52.7
Age 5–7 years	205/4672	4.4
8–10 years	1027/4672	22.0
10–12 years	3440/4672	73.6
Parental allergy ^6^	1155/4567	25.3
High parental education ^7^	1924/4531	42.5

n/N = number of observations/total number of observations;

^1 ^specific IgE against cat, mite, grass or birch >= 0.7 kU/l; ^2^specific IgE against grass or birch >= 0.7 kU/l; ^3^specific IgE against cat or mite >= 0.7 kU/l; ^4 ^total IgE >= 400 kU/l; The different sensitization denominatorsof result from different numbers of missing values.^5 ^numbers do not add up to 100% as children could be exposed to high ARI in more than one period; ^6 ^at least one parent reported a history of asthma, hay fever or eczema; ^7 ^at least one parent completed high school;

### Acute respiratory infections (ARI)

**Figure 1:**
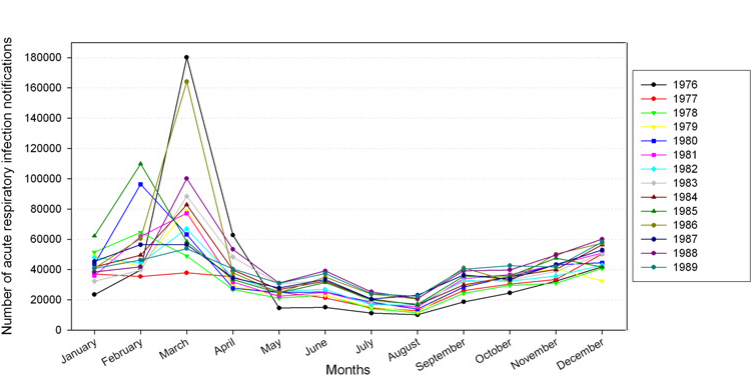
Number of acute respiratory infection notifications.

The number of ARI notifications ranged from 10124 to 180137 ARI per month with a median of 35168 (period January 1976 – December 1989). A notification number greater than 70000 (corresponding to the upper 5%) was defined high ARI. The following months were identified as high ARI months: March 1976, 1979, 1981, 1983, 1984, 1986, 1988 and February 1980 and 1985. (figure 1)

### Multivariate logistic regression analysis and polynomial distributed lag models

For multivariate logistic regression analysis confounding of the association between high ARI exposure and the atopic outcomes was tested for sex, parental allergy, parental education, area, age-group, family size and month of birth. Age-group and month of birth confounded the association between high ARI exposure and any of the atopic conditions and were therefore included in the models.

**Figure 2:**
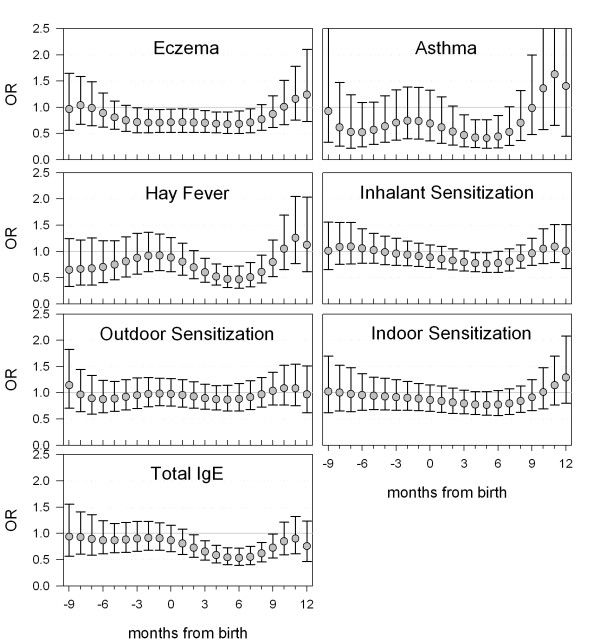
Odds ratios (OR) for the associations between exposure to high ARI and different atopic conditions. ORs have been adjusted for age group and month of birth and are shown with 95% confidence intervals.

The results from multivariate logistic regression models using disjoint exposure periods and polynomial distributed lags were similar. Therefore, we used polynomial distributed lag-models in the description of the associations between exposure to high ARI between pregnancy and the first year of life and atopic conditions at school age. Figures 2a–g show the Odds ratios of atopic conditions adjusted for age-group and month of birth. High ARI exposure within the period between the beginning of pregnancy and age one year was associated with overall reduced odds of all atopic conditions. Exposure in the first 9 months of life showed the most pronounced effect and was partly statistically significant for eczema, asthma, hay fever, inhalant sensitization and total IgE. The protective effects on inhalant sensitization seemed to be mainly influenced by the effects on indoor allergen sensitization. When the analyses were repeated for the highest 10% of ARI notifications, the effects were less pronounced, but still significant for hay fever, inhalant sensitization and total IgE for months 3–6 and additionally for total sensitization for month 6–9 and for asthma, eczema and indoor allergen sensitization for the third trimester (data not shown).

## Discussion

According to the hygiene hypothesis, infections in early life protect from allergic diseases [1]. Even though indirect evidence for this hypothesis is plenty, direct investigations of the associations between infections and atopic conditions came to conflicting results. The majority of studies measuring indirect markers of infections like siblings and day care attendance has confirmed the hypothesis [2-4]. Studies measuring past infection via IgG antibodies more consistently observed inverse associations with atopic conditions [9,10] than studies measuring clinical infections [6,11,12]. Yet, positive antibody titres only measure past immune response of the organism with the infectious agent (infection) without any indication whether clinical disease had actually taken place. Infection often represents sub- clinical infection (without symptoms) rather than clinical infectious disease (with symptoms). Likewise in our study a high risk of exposure to acute respiratory infections is not a proxy for clinical diseases but more likely a proxy for infection, a combination of sub-clinical and clinical infection. In our cohort a high pre- and post-natal risk of exposure to respiratory infectious agents was associated with decreased frequencies of atopic conditions at age 5–14 years. Interpreting the current literature and our results, we propose that the previous negative findings of studies investigating the associations between clinical infections and atopic conditions [11,12] might have been due to the fact that those studies considered solely clinical diseases whereas clinical *and *sub-clinical infection (exposure to infectious agents) in early life might protect against atopic conditions. This concept might also explain the strong and consistent findings of sibling and day care effect [2-4] as both siblings and day care increase the risk of exposure to infections rather than representing clinical infections of the children. Moreover, this concept would be in line with consistent findings of inverse associations between non-pathogen microbial stimulations (endotoxin, pro-biotics, farming environment) and atopic conditions [8,17,18].

Atopic conditions occur early in life. Therefore factors acting in early life or during pregnancy are thought to play an important role in the prevention of atopic diseases. The results of several studies indicate that exposure to factors related to the hygiene hypothesis before the first year of life confer more protection than exposures at a later age [3,4,6,8]. Responses of the immune system to specific allergens and endotoxin have been shown to start already in utero [5,7]. However, it is still not clear in which pre- or post-natal period exposure to infection is most effective in the prevention of atopic diseases. Most of the IgG studies were cross-sectional [9,10]. Therefore the time-sequence of events could not be established and methodological limitations such as recall bias were unavoidable. In our historical cohort study the observed protective effects of pre- and post-natal high risk of exposure to infectious agents on hay fever, specific and total sensitization at age 5–14 years were particularly pronounced in after birth periods. In relation to asthma and eczema this confinement was less clear. According to the current understanding early priming of the immune system might result in a lasting imbalance between Th1 and Th2 cells. Thus, it is assumed that microbial stimuli confer protection against allergies by the induction of "protective" Th1 immune responses. However, a physiological Th1 deficit exists at birth [19,20]. In addition, neonatal dendritic cells have a lower capacity to secrete IL-12, which is a key cytokine needed for the priming of Th1-responses [21,22]. Thus it is conceivable, that environmental factors may have a stronger impact in the first year of life as compared to foetal or neonatal periods. On the other hand, ARI most probably represent viral infections in the majority of cases. Therefore, it is still possible that bacterial stimuli or the combinations of bacterial and viral components or several bacterial species have a stronger impact also on foetal or neonatal immune responses. To our knowledge this is the first study that was able to examine the vulnerable period looking at both pre-natal and post-natal exposure periods.

Another unresolved issue is the type of infection which is thought to confer protection. Matricardi et al. found that inverse associations between number of older siblings and atopy were not completely mediated by hepatitis A antibodies and concluded that hepatitis A is not the only infection associated with low prevalence of atopy [9]. However, protective effects of infections have been mainly described for gastrointestinal infections [23]. In combination with observations of an increased risk of atopic diseases in children treated with antibiotics [12,24] and protective effects of pro-biotic nutrition [17] a main role of the gastrointestinal system is therefore assumed. This notion has further been fuelled by findings of positive associations between respiratory infections (e.g. respiratory syncytial virus (RSV)) and atopic diseases. Two explanations have been proposed. One assumes that RSV-infections primary damage the respiratory system. The other one sees RSV infection facilitated by primary predisposition or an already altered respiratory system [25]. However, there is also some evidence that the severity of respiratory tract infections might influence the direction of the observed effects on atopic conditions with less severe infections resulting in a decreased risk [6,26]. If lighter infections would be responsible for a protective effect on atopic diseases then our hypothesis that sub-clinical infections would also confer protection would fit. Moreover, protective effects might not be refined to gastro-intestinal infections.

We had the unique possibility to conduct a historical cohort study with notification data of the German Democratic Republic thereby avoiding shortcomings of recall bias while considering the time-sequence of events. However, potential limitations of the study have to be considered in the interpretation of our findings. Exposure to ARI is a proxy measure of individual exposure to ARI therefore measuring "risk of exposure" rather than individual exposure. This might be more problematic when considering false negative exposures. While it is likely that during the high exposure months, most children respectively mothers will be exposed to the virus, many will likely also be exposed in the lower exposure months. Even though we think that it would only affect a small proportion of our population this possible bias constitutes a potential concern. However, for the vast majority of the population our results should hold true. We measured the effect of the highest 5% and 10% of ARI notifications – where contact with infections was very likely even though clinical infections not necessarily had to occur. Thus, there was a high probability that all individuals living in the study area at that high ARI time were exposed to ARI. Non-differential misclassification would bias the effects to unity. Therefore, in case of non-differential misclassification the true underlying effects would be even larger than the observed effects. Moreover, it should be kept in mind that there is no feasible-and-ideal way of measuring individual exposure to the combination of clinical AND non-clinical infections. Our exposure assessment inhibits weaknesses but at the same time also confers some important advantages, namely that it is at the same time a proxy for non-clinical infection (similar to day care attendance and siblings). Non-clinical infection also leads to an activation of the immune system and consequently might confer some of the protective effect on allergic diseases development that has been proposed by the hygiene hypothesis. Confounding also has to be considered in the interpretation of the results. However, when testing potential confounding factors in our model, only age group and month of birth confounded the associations between exposure to ARI and any of the investigated conditions and were therefore adjusted for. It was difficult to differentiate between the effects of month of birth and high ARI. In crude analyses month of the year was statistically significantly associated with most of the atopic outcomes. However, when we adjusted for month of birth, high ARI remained statistically significant. Moreover, the observed inverse associations between high ARI and specific sensitization were mostly influenced by indoor allergen sensitization. The opposite would have been expected if month of birth as a proxy for exposure to pollen would have been the underlying causal factor. We therefore believe that the observed inverse associations between high ARI and the atopic outcomes were not entirely due to collinearity. Birth month has been discussed as a potential risk factor of allergies. However, the literature until now remained inconclusive [27]. The protective effects were also pronounced for total IgE. The interpretation for these findings is not clear to us. Individual data in our analysis were collected at age 5–14 years. Therefore, children might have had a different ARI exposure at birth time if they had moved to the study area from other regions. We had only limited information about the place of birth of the children. However, this potential kind of exposure misclassification would also have resulted in an attenuation of the effects, where the true effect would have been larger. We had no data on older siblings and used family size as a proxy variable. Nevertheless, we think that family size is a good proxy variable, because in Germany grandparents, aunts and uncles normally don't live with the family. Therefore, family size is mostly defined by the number of siblings.Multiple testing might have constituted a further limitation of our study. The analysis to identify the vulnerable period in which exposure to infection might protect from development of atopic conditions was explorative as we had no hypothesis about the exact timing of this protective effect. However, the consistency of our findings of a protective effect of months 6–9 in relation to hay fever, specific and total sensitization and the observation of slight dose response relationship argue against merely chance findings. Moreover, it seems plausible that the vulnerable period for eczema precedes the vulnerable periods for hay fever and asthma, as this is the order in which the respective clinical conditions manifest.

## Conclusion

Our results support the hygiene hypothesis and add evidence that a high risk of exposure as early as the pre- and post-natal period might be associated with decreased frequencies of atopic diseases. The post-natal period thereby seems to be particularly important. Sub-clinical infections rather than clinical infections might lead to the protective effects of infections on atopic diseases. The protective effect of infections might also be conferred by respiratory tract infections.

## Competing interests

The author(s) declare that they have no competing interests.

## Authors' contributions

AZ carried out the statistical analyses and drafted the manuscript. SK and UG carried out statistical analyses and participated in the manuscript draft. SKE participated in the drafting of the manuscript. JH participated in the design and coordination of the study and helped to draft the manuscript. All authors read and approved the final manuscript.
